# Mobile applications, physical activity, and health promotion

**DOI:** 10.1186/s12913-025-12489-z

**Published:** 2025-03-10

**Authors:** Pedro Sousa Basto, Priscila Ferreira

**Affiliations:** NIPE, EEG, University of Minho, Campus de Gualtar, Braga, 4710-057 Portugal

**Keywords:** Disease prevention, Mobile applications, Physical activity, Physical and mental health, Public health promotion

## Abstract

**Background:**

This paper studies the role of mobile applications in promoting physical activity and user loyalty to them. In doing so, our study offers fresh insights into the role of mobile applications in promoting physical activity and healthier lifestyles, filling gaps in the existing body of research.

**Methods:**

A non-probability purposive sample of adults who engage in physical exercise and use monitoring apps was selected, and semi-structured interviews were used to collect information.

**Results:**

Our findings are suggestive that (i) physical exercise is more strongly associated with the continuous use of applications than with specific loyalty strategies; (ii) widespread use of apps that record and display historical results can boost regular physical activity, as users are motivated to surpass their previous outcomes. These results support the principle that ‘more is better’ in practice and intensity, suggesting that mobile technologies should be integrated into national health plans.

**Conclusions:**

Mobile technologies should be encouraged by public policies, as these tools offer an accessible alternative for promoting public health. Policies could subsidize or facilitate the development of applications that integrate self-monitoring and personalized health plans aligned with public health guidelines. They could also include educational campaigns informing the population about these technologies’ benefits and explaining how to use them to improve physical and mental health.

**Supplementary Information:**

The online version contains supplementary material available at 10.1186/s12913-025-12489-z.

## Background

Physical inactivity is a leading risk factor for mortality related to non-communicable diseases, with insufficiently active individuals facing a 20–30% higher risk of death compared to those who are sufficiently active [1]. In 2009, physical inactivity ranked as the fourth largest global mortality risk factor, responsible for 6% of deaths, followed by overweight and obesity, which contributed to 5% [2]. Globally, in 2019, low physical activity ranked 14th in the Global Burden of Disease (GBD) among risk factors level 2, and the global age-standardized death rates of diseases attributable to low physical activity were 11.10/100,000 [3]. Due to the link between sedentary behavior and poor health outcomes, increasing regular physical activity has become a growing concern for health authorities [4].

Mobile applications have emerged as valuable tools for promoting physical activity, encouraging healthier lifestyles, and supporting users in achieving fitness goals. Prior research highlights the role of mobile health technologies in increasing health awareness, tracking exercise habits, and providing users with feedback to enhance motivation and adherence to physical activity routines. However, much of the existing literature either focuses on the technological aspects of these applications [5–8] or on user behavior in isolation [9–12]. There remains a need for research that integrates both perspectives to assess how specific app functionalities actively shape user engagement and physical activity patterns.

Our study addresses this gap by investigating how mobile applications promote physical activity and fitness goals, focusing on their impact on user engagement, the effectiveness of key app features in maintaining or improving exercise levels, and the role of loyalty mechanisms in long-term adherence. By adopting a user-centric approach, we provide deeper insights into motivations, behaviors, and experiences associated with fitness apps. Through this lens, we contribute to the literature by examining the interplay between technology and behavior, offering a real-world perspective on the practical effectiveness of mobile fitness applications and their potential role in public health promotion.

Previous studies show that physical activity, among other benefits, reduces the risk of non-communicable diseases and improves muscle mass, cardiorespiratory capacity, and mental health [13, 14]. Mobile technologies offer a cost-effective alternative to promoting population health. However, adopting mobile applications depends on social influence, perception of results, the effort required to achieve them, and behavioral intention. Understanding these variables by both populations and health regulatory entities will likely significantly impact the adoption of mobile applications to drive behavioral changes. Thus, mobile technologies can be crucial in promoting public health. However, with the widespread use of mobile devices, monitoring citizens by health authorities requires an effort toward technological integration. Studies confirm that mobile technologies offer advantages in sharing medical information between healthcare professionals and the public. For example, real-time access to health indicators allows health professionals to provide personalized activity plans to improve users’ health metrics [15, 16]. Therefore, mobile applications that manage diseases and monitor physical activity make life easier for citizens by consolidating essential information to control health and promote behavioral changes.

The effectiveness of mobile health and fitness applications is closely tied to user motivations and health concerns, as individuals who prioritize their well-being are more likely to engage with these apps consistently. Gamification features, such as challenges, rewards, and progress tracking, play a crucial role in sustaining user motivation and encouraging physical activity. These elements can be particularly beneficial for individuals who lack access to gyms, providing an engaging and structured alternative to traditional fitness environments [17]. However, user trust is critical, influencing the initial adoption and the sustained use of apps to achieve health goals [18]. Regulation of health apps can improve trust, yet it remains a challenge for authorities. For example, some AI-powered apps offer personalized health plans, but many still require regulation to ensure accuracy and prevent misinterpretation of results [19].

The Theory of Planned Behavior posits that an individual’s behavior is influenced by their attitudes, subjective norms, and perceived behavioral control [9]. When applied to mobile app adoption, this theory suggests that attitudes toward technology shape how users perceive mobile apps. These attitudes, along with other factors, influence behavioral intention. Behavioral intention is a strong predictor of actual adoption behavior. While not all studies explicitly test mediation, the consistent finding that perceived usefulness and ease of use predict behavioral intention, which in turn predicts app use, supports the notion that behavioral intention plays a crucial mediating role [5, 6, 20–22]. Sharing physical activity data online can lead to positive behavior changes through healthy competition and social interaction, though it is essential to minimize potential negative behaviors [10]. To achieve this, healthcare professionals and app developers can guide users in adopting best practices and creating engaging environments [23].

Engagement with apps through features like results monitoring, goal setting, and feedback effectively promotes behavior change, leading to higher satisfaction with app usage [11, 24]. Although incentives such as badges or trophies can motivate behavior change, excessive reliance on external rewards may reduce intrinsic motivation. To enhance efficiency, developers should focus on integrating multiple features into a single app, simplifying information gathering, providing possibilities for personalization, and ensuring app operational stability for a positive user experience that facilitates continued use of the app [24–26].

Mobile fitness apps have shown potential to influence health behaviors, but their effectiveness remains uncertain. While some studies found positive impacts on attitudes, perceived behavioral control, and physical activity levels [27, 28], others reported limited evidence of significant behavior change or health outcomes [29]. Apps commonly incorporate behavior change techniques like self-monitoring, feedback, and goal-setting [30–32], with features satisfying basic psychological needs being crucial for perceived usefulness and usage [33]. However, concerns exist regarding the potential for apps to foster maladaptive eating and exercise behaviors, particularly among young people [34]. User perceptions of apps are generally positive, with high engagement and ease-of-use ratings reported [29]. To improve effectiveness, researchers suggest incorporating behavior change theories in app development and focusing on usefulness and perceived difficulties [28, 35].

User loyalty in exercise apps refers to the sustained and consistent use of an app over time, reflecting a commitment to its features and services. This includes frequent logins, continuous goal tracking, participation in challenges, and renewal of paid subscriptions (if applicable). Loyalty can be measured through user retention rates, session frequency, and adherence to personalized workout plans. However, sustained engagement with fitness apps depends on factors such as trust, perceived usefulness, social influence, and users’ digital lifestyles [7, 8, 36, 37]. Understanding users’ digital behaviors can improve communication and usability. Perceived behavioral control, a key predictor of physical activity, reflects an individual’s determination to continue using an app, making credibility and social connectivity essential for long-term retention [38]. Additionally, apps with a simple, customizable design and a positive user experience are more likely to foster lasting engagement [39].

Maintaining user loyalty to mobile apps is challenging due to competition among apps and users seeking new challenges [12]. Developers can enhance loyalty by incorporating goal setting, continuous monitoring, and feedback. Research on fitness apps highlights several trends related to retention and long-term adherence. For example: (i) gamification is a popular strategy that applies game mechanics to non-gaming activities, promoting behavior change and physical activity [40, 41]. Studies show that gamification elements (e.g., point systems, leaderboards) significantly improve user engagement and adherence [42, 43]; (ii) users who integrate app usage into their daily routine (e.g., setting reminders, using streak-based incentives) are more likely to sustain engagement [32]; (iii) many users abandon fitness apps within the first few weeks due to lack of motivation, poor user experience, or unrealistic expectations [44]; (iv) apps with social features, such as group challenges or peer encouragement, see higher retention rates compared to solitary-use apps [45]; and (v) apps grounded in psychological theories of behavior change, demonstrate higher effectiveness in sustaining long-term usage [46].

Technology content developers and marketers are crucial in creating personalized apps that meet user needs. Understanding user intentions and designing simple, engaging, and valuable apps is critical to their success [6]. Developers must consider user preferences, including gender differences, and work towards enhancing performance, reducing effort, and addressing privacy concerns, particularly for users with chronic conditions [7, 47]. Fitness apps should deliver value by optimizing performance, ensuring privacy, and building trust while collaborating with developers, marketers, and government authorities to empower users to manage their health effectively [48]. While gamification is widely used in fitness apps to keep users engaged and motivated, developers must ensure that the app remains challenging and avoids over-reliance on external rewards [49]. Effective gamification strategies should focus on achievable goals and motivating challenges to enhance user retention and promote long-term engagement.

Digital technologies can foster better communication between marketers and stakeholders, enabling co-creation and stronger partnerships. Understanding user behavior with mobile apps and identifying the most effective retention strategies– such as self-monitoring, gamification, and fostering group belonging– is valuable for stakeholders aiming to encourage physical activity through technology.

The paper is organized as follows. The next section describes the empirical strategy. Section 3 presents the results. Section 4 discusses policy implications. Section 5 concludes.

## Methods

### Data collection

We study the use of health and fitness apps to promote physical activity and loyalty methods concerning these apps. Several steps were taken to minimize potential biases in data collection. We conducted semi-structured open-ended interviews, known for their flexibility, with interviewers adjusting questions as the conversation evolved. This enabled a deeper understanding of the subject and rich data collection, reducing interviewer bias. This qualitative-interpretative approach requires participant involvement and the researcher as an impartial narrator.

A nonprobability purposive sample was used to select participants based on specific criteria– engagement in physical exercise and use of digital monitoring apps–which helped to mitigate selection bias. The study included 37 participants (28 women and nine men), ensuring data saturation and minimizing sampling bias within the recommended range for semi-structured interviews [50, 51]. Snowball sampling, though prone to bias, was chosen due to the lack of data on the population of digital app users in physical exercise, making random sampling impractical. Initial contacts, primarily university students who responded to a university–wide email invitation, along with colleagues who fit the research criteria, were invited to participate. Those who agreed were encouraged to refer others who also met the requirements. Participants provided informed consent after being briefed on the study’s purpose, participation risks (none), benefits of research owing to their participation, and data protection measures, reducing response bias. Ensuring participant anonymity helped mitigate social desirability bias. The denaturalized transcription technique maintained data accuracy by omitting nonverbal elements and speech irregularities. Including participants aged 19 to 66 helped reduce age-related bias.

The interview script (available in the Supplementary Materials) was constructed considering the existing literature and the specific objectives of our research: (1) Identify the motivations to use mobile applications in exercise monitoring (SO 1); and (2) Identify the best strategy to retain app users, ensure the sustainability of the application and thus enhance the probability of being physically active (SO 2). Interviews were conducted via videoconference.

### Rationale for the chosen design and analysis process

This study employs a qualitative-interpretative approach using semi-structured open-ended interviews to explore how health and fitness apps promote physical activity and retain users. This design was chosen to capture in-depth insights into user motivations and behaviors that are not easily quantifiable. Given the study’s exploratory nature, content analysis was selected as the primary method of data analysis for its ability to identify patterns and themes within qualitative data systematically.

Content analysis is particularly suitable for this study as it allows for a structured examination of qualitative data by identifying commonalities and variations in participants’ responses. It facilitates theme development aligned with the study’s objectives: (i) motivations for using mobile exercise applications and (ii) strategies for user retention and app sustainability. Content analysis also provides a flexible yet systematic approach that accommodates the richness of open-ended interviews while maintaining analytical rigor. Additionally, it complements existing quantitative research on fitness app usage by offering deeper insights into user perspectives.

### Analysis process and steps

The analysis followed a systematic process to ensure rigor and depth. Interviews were conducted via videoconference, allowing interviewers to adapt questions based on participant responses, promoting richer insights. Given the semi-structured nature of our interviews, we did not impose a fixed duration to allow participants the flexibility to provide detailed responses. For clarity, all interviews were conducted in Portuguese. The denaturalized transcription technique was applied, omitting nonverbal elements and speech irregularities to focus on meaning and content. Researchers thoroughly reviewed transcripts, making preliminary notes to identify emerging themes and recurring patterns. An inductive coding approach allowed themes to emerge naturally rather than being predetermined, with codes grouped into meaningful categories aligned with the study objectives. The results were synthesized to highlight key factors influencing fitness app adoption and retention strategies. To enhance visualization, we incorporated word clouds and transcribed select excerpts from different interviews. Some quotes were assigned to multiple categories when they meaningfully contributed to different themes. Finally, the identified themes and patterns were systematically linked to the research questions and theoretical frameworks to ensure coherence and analytical depth.

### Ensuring trustworthiness in the analysis

Several strategies were implemented to enhance credibility, dependability, transferability, and confirmability. Semi-structured interviews provided flexibility, allowing for deeper exploration of the subject, and the interviewer ensured in-depth conversations to capture rich data. Participants were informed about the study’s purpose and data protection measures, enhancing ethical conduct and trustworthiness, and were allowed to review transcripts for accuracy. Researchers acknowledged their role in shaping the interpretation of data, ensuring impartiality and minimizing bias. Colleagues reviewed findings to challenge potential biases, further ensuring robustness.

By employing this structured qualitative analysis approach and ensuring trustworthiness through rigorous methods, the study maintains the rigor and reliability necessary for high-quality qualitative research while providing valuable insights into digital fitness app usage and retention strategies. Despite measures taken to mitigate biases, some limitations remain due to the nature of qualitative research and the sampling method used.

## Results

WHO’s Global Action Plan on Physical Activity 2018–2030 reads: “*There are many ways to be active– walking*,* cycling*,* sport*,* active recreation*,* dance*,* and play - and many policy opportunities to increase participation.*” ([52], p.1). Building on this framework, our study explores how mobile fitness apps can serve as effective tools to increase physical activity both extensively (by encouraging more individuals to become active) and intensively (by promoting more frequent and sustained engagement).

In the following subsections, we present our findings across three key areas. First, we analyze physical activity patterns, including the diversity of activities, frequency, and motivations for engaging in exercise. Next, we explore the role of mobile apps in shaping exercise behaviors, with a focus on self-monitoring, key app features, and the influence of tracking health outcomes. Finally, we assess the impact of gamification and social engagement strategies on app retention and sustained physical activity. Together, these insights provide a comprehensive understanding of how digital fitness tools can promote and support long-term physical activity.

### Physical activity patterns: diversity, frequency and motivations

**Fig. 1:**
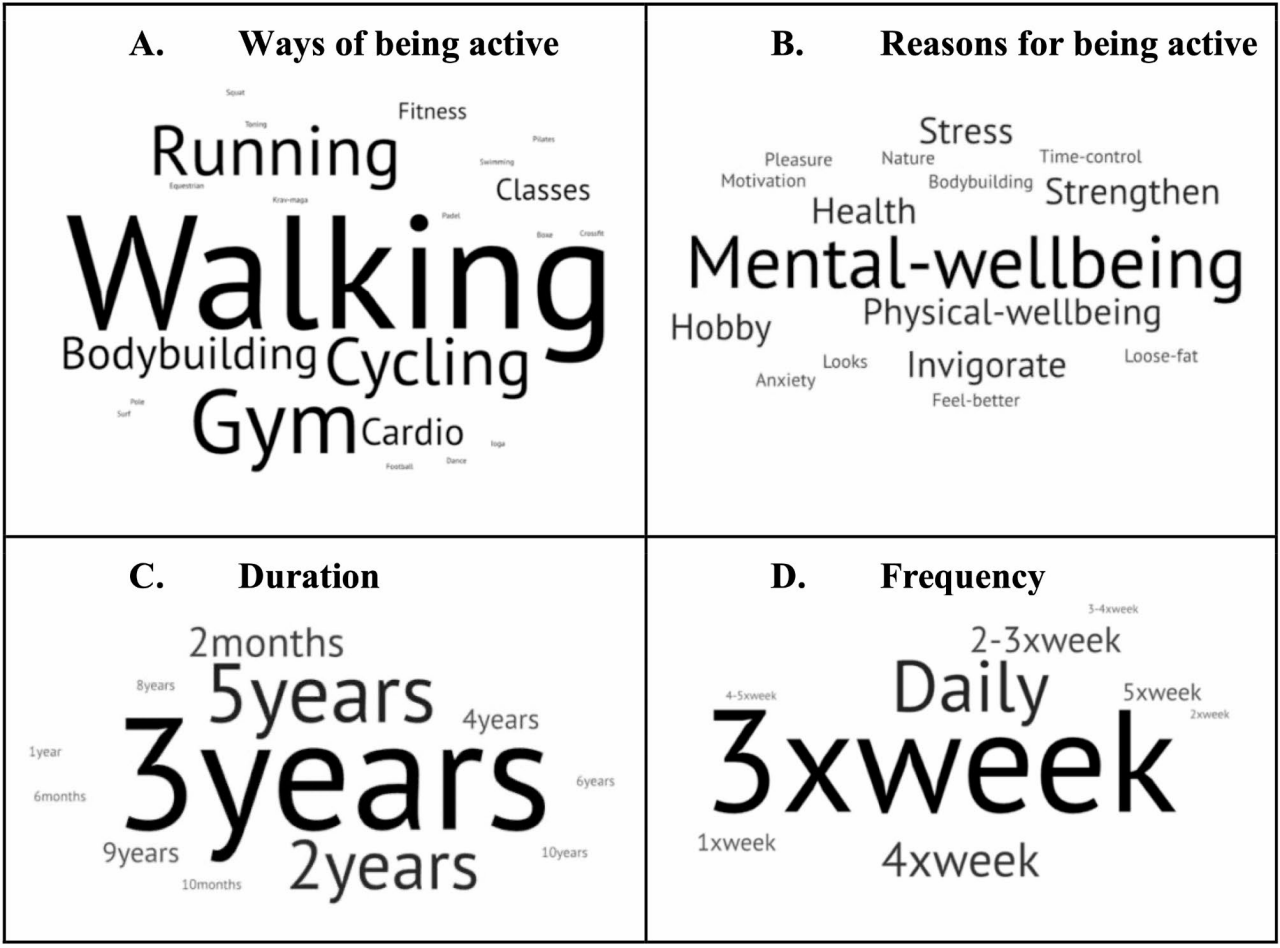
Characterizing physical activity, sample results

Understanding the patterns, motivations, and frequency for the practice of physical activity is crucial for developing effective physical activity interventions, especially for diverse populations. People engage in a wide range of physical activities. Among the 22 different activities named by the participants in our study, the five most common responses were walking (48%), going to the gym (27%), running (21%), cycling (21%), and bodybuilding (18%). Although some examples may overlap in content, our results suggest that participants in our sample understand that one can be active in several ways. In Fig. 1, Panel A, we depict a word cloud of these results.

The most common reasons for engaging in physical activity (Fig. 1, Panel B) are mental and physical well-being, as sports aid in managing anxiety and stress and help prevent and treat non-communicable diseases. When examining for how long (duration) and how often (frequency) individuals engage in physical exercise (Fig. 1, Panels C and D, respectively), we observe some began exercising regularly at least five years ago (18%), three years ago (21%), and two years ago (15%). This led us to suspect that periodic physical exercise routines may have changed due to the lockdown periods imposed during the pandemic. The most common frequencies individuals exercise are three times a week (36%), daily (18%), and four times a week (12%).

**Fig. 2:**
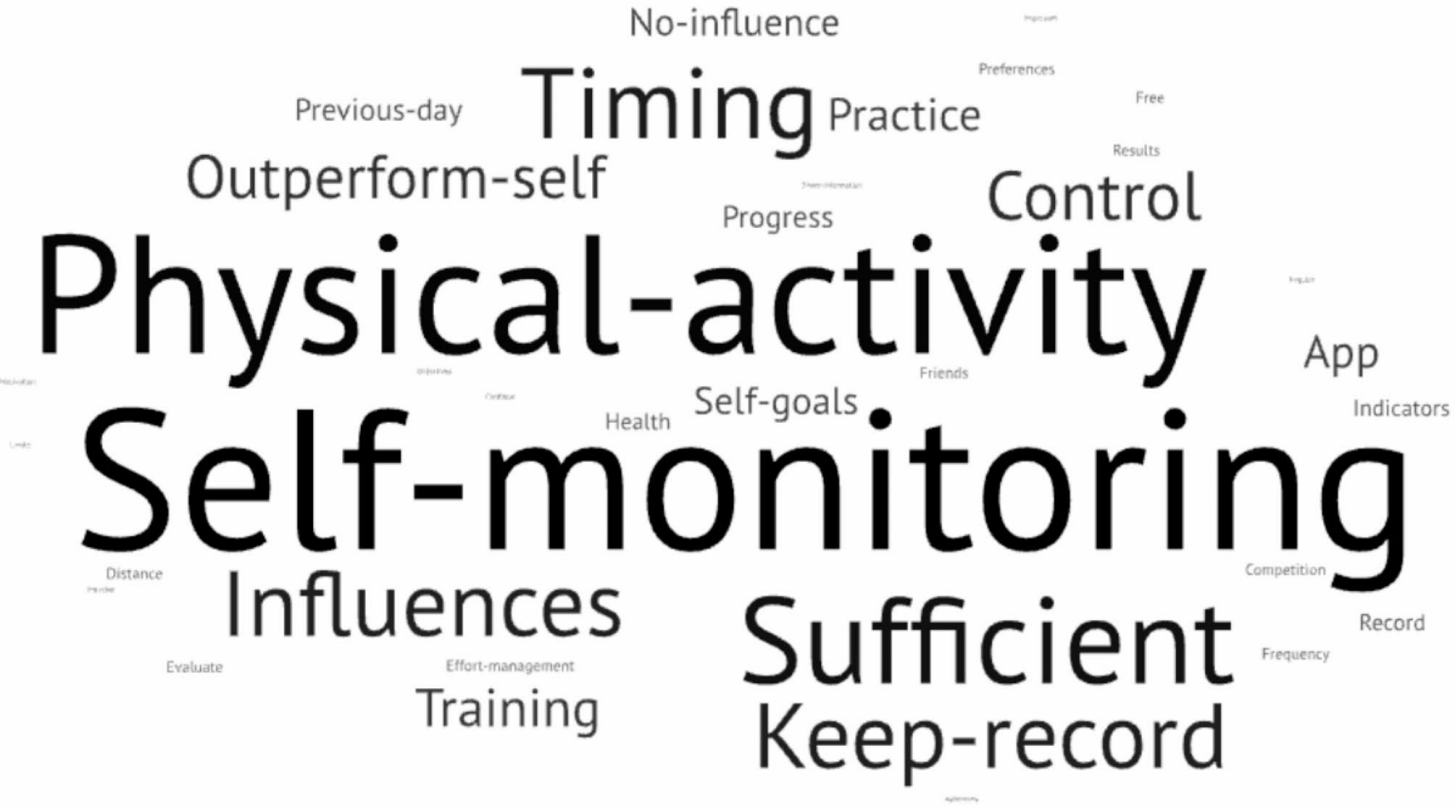
Motivations to use apps

Figure 2 depicts a word cloud emphasizing the most common reasons for using mobile apps. Self-monitoring is the most frequent reason (79% of respondents) for using mobile fitness apps when engaging in physical activity. Excerpts of some of the responses we obtained can be read in Table 1.

**Table 1:** Motivations to use apps

“Comparing walking time across different weeks. Knowing how much I’ve walked. Self-monitoring influences exercise.” (interview #12).
“Evaluation of training and comparison of results. Self-monitoring is sufficient for me to engage in exercise.” (interview #27).
“I personally enjoy monitoring exercises and physiological responses; it also serves as a social reference and allows us to share with friends and see other people’s activities. Self-monitoring of indicators is sufficient for exercising.” (interview #35).

The main motivations for using mobile applications for self-monitoring in physical activity include tracking progress, assess training performance and monitor improvements, and self-motivation. Overall, self-monitoring enhances awareness, accountability, and consistency in exercise routines.

### Mobile app utilization: impact, and features in physical activity

In this section we discuss the multifaceted role of mobile apps in promoting and supporting physical activity. The key parameters of interest for users when monitoring physical activity include: performance metrics, effort management, comparison over time, cardio indicators, usability, that is, users show preference for practical, intuitive, and free apps that guide activity. Overall, users value data that helps them stay focused on goals, manage effort, and track improvements (See Table 2).

**Table 2:** Parameters of interest

“Monitoring speed, heart rate, stride length, etc., is a good way to stay focused on goals, manage the effort spent throughout the day and during workouts, as the app allows for comparison of walking time across different weeks, as well as the distance covered.” (interview #4).
“Practical, intuitive, and free. Related to professional preferences. Controls appropriate activity and guides walks, as well as an understanding of cardio indicators.” (interview #2).
“Due to personal preference. Management of the effort expended throughout the day and during workouts.” (interview #13).
“Control of physical activity. Free app.” (interview #8).

Our interviews further suggest that physical activity is enhanced by maintaining a record of past results, enabling users to manage their routines more effectively. Keeping records increases individuals’ awareness of their physical activity, encourages commitment to regular exercise, allows them to assess progress, and challenges them to surpass their previous achievements (See Table 3). In summary, data provided by mobile apps that reflect individual performances can serve as a motivation to continue being active. Our results also suggest that the ease of scheduling classes, evaluating training, and comparing results can create a dependency, imposing a mandatory routine on the user. That is, people use apps to stay active. Additionally, the functionality and applicability of these apps are not limited to monitoring sports practice. Responses indicate a direct link between the use of these applications and overall health, as evidenced by the use of apps that help monitor daily physical exercise and goals’ achievement such as maintaining weight, losing weight, or increasing muscle mass.

**Table 3:** On the importance of keeping a record

“Recording physical activity. Self-monitoring is sufficient for me to engage in physical exercise. Having a history of completed activities.” (interview #37).
“Progression and surpassing times. Self-monitoring is sufficient for me to engage in physical exercise and influences my exercise practice.” (interview #28).
“Monitoring and focusing on goals. Self-monitoring is sufficient for physical exercise and influences the duration of exercise practice. Monitoring and tracking progress.” (interview #19).
“Monitoring workouts and physical indices. Self-monitoring is sufficient for me to engage in physical exercise and influences the amount of time I spend exercising. The use of the app makes exercise feel mandatory.” (interview #36).
“Tracking training statistics. Self-monitoring is sufficient for me to engage in physical exercise. My exercise practice is for my well-being and satisfaction; other factors do not influence me.” (interview #32).

Results from our sample underline the importance of tracking results for supporting physical activity and confirm that many participants are concerned with the health indicators provided by their device. Overall, tracking health outcomes enhances awareness, safety, and motivation in maintaining an active lifestyle. (See Table 4).

**Table 4:** On the importance of tracking health outcomes

“I find it interesting to access my data and compare it with the different sports I practice, such as running or stretching, and see how my heart rate varies.” (interview #21).
“Monitoring heart rate, caloric intake, and distance covered. Self-monitoring is sufficient for engaging in physical exercise.” (interview #23).
“Weightlifting and cardio because I genuinely enjoy exercising for the sake of mental well-being.” (interview #28).
“Recording physical activity. I use the app because it benefits my health. It helps me know if my heart is working properly or not, and if I’m close to reaching my physical limits.” (interview #30).

Being able to exercise without a fixed schedule (as opposed to attending gym group classes, e.g.) is also one of the reasons pointed out why monitoring apps are used (See Table 5).

**Table 5:** On the importance of flexibility

“I have difficulty attending the gym due to scheduling conflicts and prefer to exercise independently and at my own pace. The apps have reminders that alert me to the need to exercise and allow for permanent data recording and a summary of monthly statistics.” (interview #25)	

### Gaming elements in fitness apps: strategies, motivation, and social dynamics

**Fig. 3:**
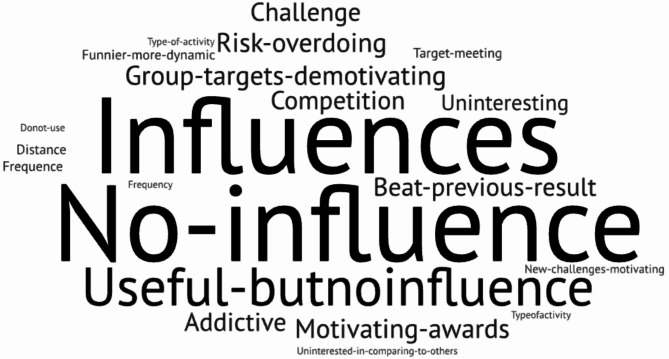
Gaming influence on app usage and sports practice

The influence of gaming strategies on mobile fitness app usage and physical activity levels is complex and varies among users. Based on our results, we can identify several key points. A recurrent factor that incentivizes the sustained use of a mobile application is the personal historical record and the will to outperform the previous day’s outcomes. This overcoming, however, can be achieved in two ways: (i) individual surpassing of personal goals, depending solely on one’s effort, commitment, and dedication; or (ii) in a gaming context with challenges and competitions and where goals are set in comparison with other members. In Fig. 3, we depict a word cloud concerning the influence of gaming strategy on app usage and, thus, being physically active. Results are not clear cut as the balance of responses is striking. Some say gaming strategies influence their app usage, while others do not. The varied responses in our word cloud reflect the complexity of user motivations and the need for diverse strategies in fitness apps to cater to different user preferences and motivational factors. Among the reasons pointed out for a gaming environment not being a determining factor for their practice is that group targets can be demotivating, negatively impact respondents’ self-esteem, and eventually become hazardous (See Table 6).

**Table 6:** On the risks of keeping up with the Joneses

“Comparing with other members is demotivating. We can fall into the trap of trying to achieve goals that go beyond our physical limits, which could harm us.” (interview #1)	

Other mobile app users have shown interest in the gaming environment, thus suggesting that gaming strategies can extend user loyalty to the app. The gaming environment creates a need for individual achievement among members, considering the higher indicators of the group. Challenges and competition are highly addictive and make the process more fun and dynamic, encouraging participants in exercise practices, types of activities, and frequency (See Table 7).

**Table 7:** On the influences of gaming strategies

“Challenge and competition (very addictive)” (interview #11)
“Games make the process more fun and dynamic.” (interview #14)
“The gaming environment influences me because I always try to reach goals.” (interview #15)
“It influences my frequency and distance.” (interview #16)
“Creating challenges motivates users to explore these activities, even if just out of curiosity.” (interview #26)
“Challenges influence the type of activity and frequency.” (interview #34)

Overall, gaming elements in fitness apps appear to have a significant positive impact on user engagement, motivation, and exercise habits, making fitness activities more appealing and goal-oriented.

## Discussion and policy implications

The literature highlights the crucial role of mobile technologies in promoting health by helping users achieve optimal health metrics and adopt healthier lifestyles, given their growing health awareness. Our findings align with previous research, demonstrating that intrinsic motivations– such as the need for autonomy, competence, and relatedness– positively influence attitudes toward exercise, improve physical indicators, and enhance self-monitoring behaviors [33]. Notably, individuals who actively track their physical activity are more likely to maintain regular exercise routines. While ease of use and trust influence the initial adoption of fitness apps [5, 36], long-term engagement is primarily driven by self-monitoring and goal-setting. Overall, mobile apps contribute to healthier lifestyles by fostering consistent physical activity and better daily habits, aligning with WHO guidelines on physical activity [4].

However, our study has some limitations that should be considered when interpreting these findings. First, our sample may not fully represent all user demographics, particularly individuals who do not engage with fitness apps or those from lower-income backgrounds who may face barriers to technology access. Future research should explore adoption barriers among non-users to gain a more comprehensive understanding of mobile fitness app accessibility. Second, the cross-sectional nature of our study prevents us from assessing long-term engagement trends, limiting our ability to evaluate whether certain app features maintain user interest over extended periods. Longitudinal studies would help clarify the sustainability of app engagement over time.

Our findings have significant public policy implications for promoting physical activity and health. Governments should prioritize leveraging mobile technologies as public health tools. The widespread use of mobile health technologies during the COVID-19 pandemic highlights the effectiveness of integrating technology into health promotion. Therefore, policymakers should actively promote mobile technologies as accessible and inclusive tools for public health enhancement. However, while mobile fitness apps can potentially improve public health, their accessibility is shaped by factors such as socioeconomic status, digital literacy, and access to technology. Policymakers must consider these disparities to ensure that mobile health initiatives are equitably available to all populations.

To maximize their impact, public policies could support the development of fitness apps that integrate self-monitoring and personalized features aligned with public health guidelines, potentially through subsidies or incentives to enhance accessibility. Centralized health monitoring platforms could facilitate data-sharing between users and healthcare professionals, enabling tailored physical activity plans. Additionally, educational campaigns should raise awareness of mobile fitness apps’ benefits and promote their appropriate use for improving physical and mental well-being. Since self-monitoring is a key driver of sustained app engagement, public policies should support the development of technologies that promote self-improvement through historical activity tracking [19].

A key limitation of fitness apps is the lack of professional supervision, which underscores the importance of human factors in exercise adherence. While apps provide automated feedback, they may not fully replace the personalized guidance trainers offer, which could impact user safety and the effectiveness of training programs. To address this gap, policymakers could introduce certification programs or regulatory measures to ensure fitness apps adhere to health and safety standards, minimizing potential risks to users. Additionally, fitness app developers and marketers should focus on enhancing user engagement through personalization– especially historical data tracking– which has proven to be a critical factor in retention [17].

One of our key objectives was to identify the most effective retention strategies for prolonged app use. Our findings suggest that self-surpassing and historical performance tracking are sufficient to sustain user engagement. However, our study had limitations in evaluating the impact of gamification on app loyalty, possibly due to our predominantly adult-focused sample. Future research could explore this aspect with younger demographics, who may respond differently to gaming elements in fitness apps [52]. Moreover, disparities in smartphone access, internet reliability, and digital literacy limit the ability of some individuals to benefit from these tools fully. This raises important equity concerns, as mobile health technologies may not be equally accessible to all populations. Investigating these barriers could provide valuable insights for designing more inclusive and engaging fitness technologies that better serve diverse user needs.

## Conclusions

Our study confirms that individuals engage in physical activity in various ways, with walking, gym workouts, running, cycling, and bodybuilding being the most common. This aligns with WHO’s *Global Action Plan on Physical Activity (2018–2030)*, which emphasizes the importance of providing varied opportunities to stay active. The primary motivations for exercise among respondents relate to mental and physical well-being, highlighting the role of physical activity in managing stress, reducing anxiety, and preventing non-communicable diseases, which are major contributors to the global burden of disease [2, 3].

Our findings contribute significantly to the existing literature by demonstrating that sustained app usage is more strongly linked to physical exercise than to specific loyalty strategies. Notably, mobile fitness apps that record and display historical performance data can play a crucial role in reinforcing regular physical activity, as users are motivated to surpass their previous achievements. Self-monitoring– particularly through tracking personal progress– emerges as the most effective strategy for maintaining engagement with these apps and fostering long-term exercise habits [44].

By adopting a user-centric perspective, this study offers deeper insights into how individuals interact with mobile fitness applications, emphasizing their motivations, behaviors, and experiences. This approach underscores a key aspect of our analysis: the intersection between technology and behavior. Our findings suggest that mobile fitness apps serve as powerful tools for promoting physical activity by facilitating habit formation, motivation, and self-regulation [32, 48]. Given their broader role in health management, future research could explore how these digital tools can further enhance user commitment and overall well-being [45, 46].

In conclusion, our research provides valuable insights for policymakers, app developers, and health professionals seeking to leverage mobile technologies for promoting physical activity and healthier lifestyles. By prioritizing self-monitoring, personalization, and responsible app use, we can maximize the potential of these technologies to support long-term engagement in physical activity and overall well-being.

## Supplementary Information

Supplementary Material 1

## Data Availability

Qualitative data. Some excerpts from the interviews were provided in the main text, and organized according to the issue in question. Tables 1, 2, 3, 4, 5, 6 and 7.
