# Supplementary material for: Mobile applications, physical activity, and health promotion
Source: BMC Health Serv Res. 2025 Mar 10;25:359. doi: 10.1186/s12913-025-12489-z (PMC11892298; doi:10.1186/s12913-025-12489-z)
Supplement: Supplementary file 1 — Supplementary Material 1 [file 12913_2025_12489_MOESM1_ESM.docx]

# Supplementary Material

# Appendix A - Interview outline

1. Do you exercise?
2. What type of exercise do you practice and why?
3. Do you use mobile apps to monitor your physical exercise? Which ones?

**If you answered "no" to question 3, your interview ends here.**

1. What is your age?
2. What is your gender?
3. How often do you exercise?
4. How long have you been using mobile apps to monitor your physical activity? (SO 1)
5. What motivates you to use mobile apps? (SO 1)
6. Does real-time monitoring of exercise indicators influence your exercise routine? (SO 2)
7. Does the use of mobile apps influence the time you spend exercising? (SO 2)
8. In your opinion, is there any hazard in using fitness mobile apps? If so, what is it? (SO 1)
9. Have you ever felt the need to stop using the exercise monitoring app? (SO 1)
10. What motivated you to stop using the app? (SO 1)
11. Is self-monitoring of physical activity indicators sufficient to continue using the mobile app? (SO 2)
12. Is your exercise routine influenced by participants gathering in communities and comparing individual indicators? In what way? E.g., frequency, intensity, or type of activity? (SO 1 & 2)
13. Do you believe that using games and challenges extends the use of the app? How does it influence your use? (SO & 2)
14. What motivates you to use the mobile app? (SO 1 & 2)
15. Have you ever switched mobile apps for monitoring? Why? (SO 1 & 2)

**Please note: **SO** stands for **S**pecific research **O**bjective
